# Inositol 1,4,5- Trisphosphate Receptor Function in *Drosophila* Insulin Producing Cells

**DOI:** 10.1371/journal.pone.0006652

**Published:** 2009-08-14

**Authors:** Neha Agrawal, Nisha Padmanabhan, Gaiti Hasan

**Affiliations:** National Centre for Biological Sciences, Tata Institute of Fundamental Research, Bangalore, Karnataka, India; Johns Hopkins School of Medicine, United States of America

## Abstract

The Inositol 1,4,5- trisphosphate receptor (InsP_3_R) is an intracellular ligand gated channel that releases calcium from intracellular stores in response to extracellular signals. To identify and understand physiological processes and behavior that depends on the InsP_3_ signaling pathway at a systemic level, we are studying *Drosophila* mutants for the InsP_3_R (*itpr*) gene. Here, we show that growth defects precede larval lethality and both are a consequence of the inability to feed normally. Moreover, restoring InsP_3_R function in insulin producing cells (IPCs) in the larval brain rescues the feeding deficit, growth and lethality in the *itpr* mutants to a significant extent. We have previously demonstrated a critical requirement for InsP_3_R activity in neuronal cells, specifically in aminergic interneurons, for larval viability. Processes from the IPCs and aminergic domain are closely apposed in the third instar larval brain with no visible cellular overlap. Ubiquitous depletion of *itpr* by dsRNA results in feeding deficits leading to larval lethality similar to the *itpr* mutant phenotype. However, when *itpr* is depleted specifically in IPCs or aminergic neurons, the larvae are viable. These data support a model where InsP_3_R activity in non-overlapping neuronal domains independently rescues larval *itpr* phenotypes by non-cell autonomous mechanisms.

## Introduction

Calcium is a versatile signaling molecule that has been found to regulate a multitude of processes, from fertilization to cell death. The regulation of such diverse processes depends on the intricate regulation of calcium levels by an extensive toolkit that consists of calcium channels and pumps on the plasma membrane and the membrane of intracellular stores that help in assembling signaling systems with very different temporal and spatial dynamics [1]. An important component of this toolkit is the Inositol 1,4,5- trisphosphate receptor (InsP_3_R), a ligand gated calcium channel, which releases calcium from intracellular stores into the cytoplasm upon cell surface receptor stimulation. It is known that InsP_3_R is widely expressed and its role in various cellular processes has been identified using *in vitro* studies [2]. However, InsP_3_R function in the context of whole organism physiology is not well understood.

*Drosophila melanogaster*, a model system amenable to genetic and physiological manipulations, has therefore been utilized to understand both systemic and cellular requirements for the InsP_3_R [3]–[5]. Genetic analysis that ascribes genes to physiological processes however needs to be further complemented by an elucidation of the cells where these genes are functionally required. Experiments where the wild-type gene is expressed in different cellular subsets in an otherwise mutant animal to rescue mutant phenotypes help in identifying cellular components where InsP_3_R activity could underlie a physiological output. By this process, we have previously demonstrated that InsP_3_R expression in the neuronal domain and specifically the aminergic interneurons (with the *DdcGAL4*) rescues larval viability [4].

In this study, we show that larval *itpr* mutant phenotypes can be significantly rescued by restoring InsP_3_R activity in insulin producing cells (IPCs) with use of the *Dilp2GAL4* [6]. Moreover, we find that growth defects and associated larval lethality in *itpr* mutants arise as a consequence of disrupted feeding behavior. An independent requirement of InsP_3_R activity in the prothoracic gland cells that synthesize and secrete the insect molting hormone ecdysone also exists. The *Dilp2GAL4* and *DdcGAL4* expression domains do not exhibit any obvious overlap suggesting that the *Dilp2GAL4* rescue is mediated by a non-cell autonomous mechanism.

## Results

### Rescue of larval growth and viability in *itpr* mutants by restoring *itpr* function in insulin producing cells

**Figure 1:**
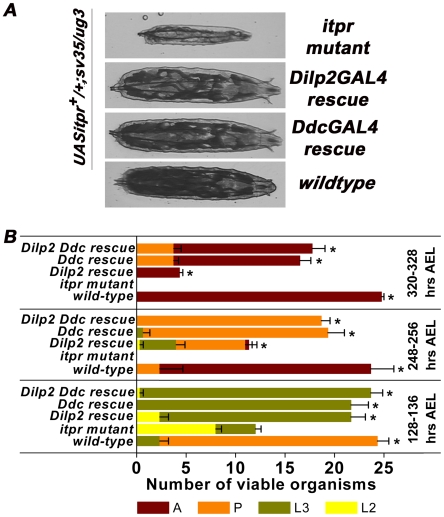
Growth and lethality defects in *itpr* mutant larvae can be rescued by *UASitpr^+^* expression in IPCs. (A) Third instar larvae at 120 hrs After Egg Laying (AEL). *UASitpr^+^/+;itpr^sv35/ug3^* are significantly reduced in size and fat body content. Both *Dilp2GAL4* and *DdcGAL4* rescued larvae start wandering at this stage and appear similar to wild-type (Canton-S) controls. (B) Wild-type (*Canton-S*) and animals of all rescue conditions grown at 25°C survive better as compared to *itpr* mutants (*UASitpr^+^/+;itpr^sv35/ug3^*) grown under the same conditions at later times after egg laying (*p<0.05; Student's t-test). For each time interval and genotype, 75 animals were screened in 3 batches of 25. Each bar represents the total viability of the indicated genotype. The colored subdivisions in each bar represent the number of larvae developing to later larval, pupal (P) or adult (A) stages. The survival profile of *DdcGAL4* rescued animals (*UASitpr^+^/+*; *DdcGAL4/+*; *itpr^sv35/ug3^*) is better than that of *Dilp2GAL4* rescued condition (*UASitpr^+^/+*; *Dilp2GAL4/+*; *itpr^sv35/ug3^*). Expression of *UASitpr^+^* simultaneously with *Dilp2GAL4* and *DdcGAL4* (*UASitpr^+^/+*; *Dilp2GAL4/DdcGAL4*; *itpr^sv35/ug3^*) does not improve survival beyond that observed with only *DdcGAL4*. Results are expressed as mean±SEM.

Mutants in the *Drosophila itpr* gene exhibit larval and adult phenotypes based on the strength of the heteroallelic combination. Stronger mutant combinations are larval lethal while adult viable combinations exhibit defective wing posture with reduced flight ability and altered flight physiology [4], [5]. Amongst the stronger allelic combinations, lethality in *itpr^sv35/ug3^* has been well characterized; a majority of these larvae die as second instars with a slightly extended lethality profile as compared with *itpr* null organisms [4]. *itpr^ug3^* is a hypomorph in which the single point mutation lies in the N-terminal ligand binding domain while *itpr^sv35^* is a null allele with a stop codon in the modulatory domain [4]. *itpr^sv35/ug3^* larvae are smaller in size as compared to wild-type controls (Figure 1). As growth in *Drosophila* is largely regulated by the insulin signaling pathway [7], the effect of restoring *itpr* function in IPCs in the brain was assessed on the growth of *itpr^sv35/ug3^* animals. The *Dilp2GAL4* strain that expresses in larval and adult IPCs [6] was utilized for expressing the wild-type *itpr* transgene (*UASitpr^+^*) in the background of *itpr^sv35/ug3^*. A significant rescue of larval size was observed (Figure 1A). About half the surviving larvae could pupate and emerge as adults in the *Dilp2GAL4* rescued condition unlike *itpr^sv35/ug3^* (Figure 1B). Growth and lethality in *itpr* mutant larvae can thus be partially but significantly rescued by expression of *UASitpr^+^* in the *Dilp2GAL4* domain.

A comparable rescue of size was also observed in *DdcGAL4* rescued animals, in agreement with previous observations where lethality of *itpr^sv35/ug3^* could be rescued by *UASitpr^+^* expression in aminergic cells [4]. The extent of rescue of lethality in *itpr^sv35/ug3^* with *Dilp2GAL4::UASitpr^+^* was less compared to *DdcGAL4::UASitpr^+^* (Figure 1B). An independent requirement of *itpr* activity in the two neuronal subgroups predicts that the level of rescue observed by simultaneously expressing *UASitpr^+^* in both *Ddc* and *Dilp2GAL4* domains should be enhanced as compared with rescue by expression in individual domains. However, rescue of pupae and adults was not significantly improved by *UASitpr^+^* expression in both *DdcGAL4* and *Dilp2GAL4* domains as compared with rescue from the *DdcGAL4* domain alone (Figure 1B). This shows that the rescue of lethality is not a simplistic summation of restoring *itpr* activity in two independent cellular domains and suggests that aminergic neurons and IPCs might communicate with each other.

### Reduced growth of *itpr^sv35/ug3^* arises from defective feeding

**Figure 2:**
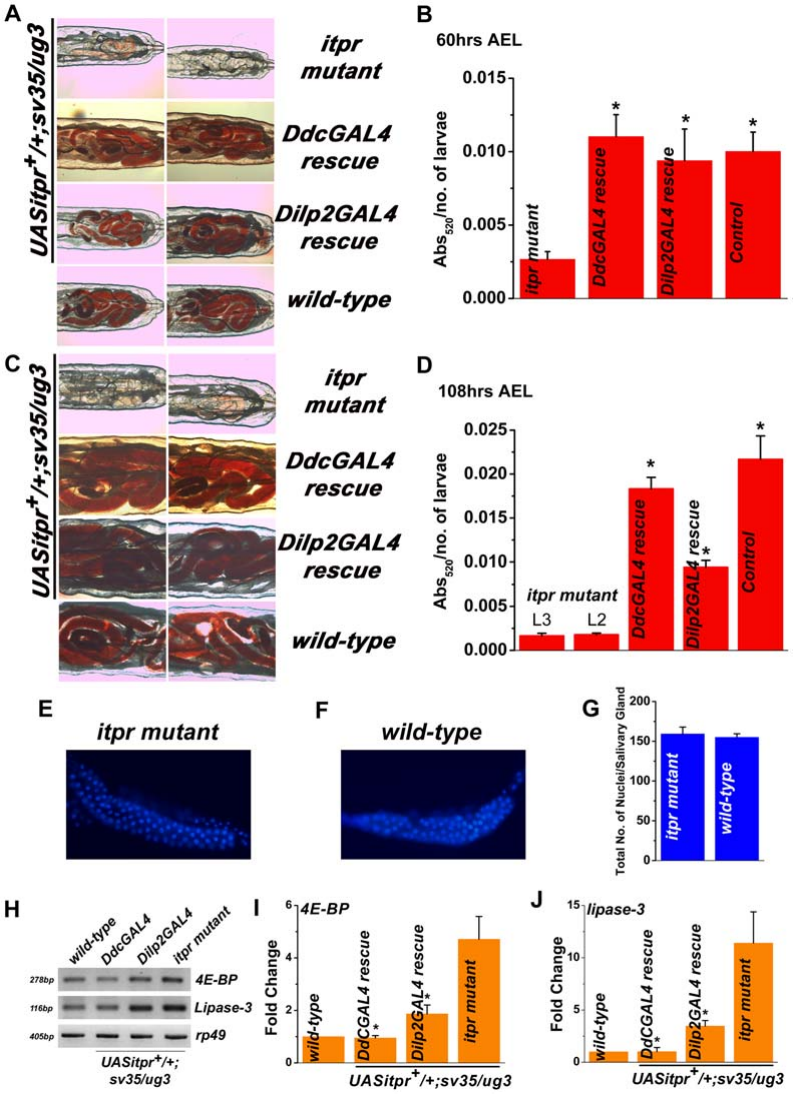
Feeding defects in *itpr^sv35/ug3^* can be rescued by *UASitpr^+^* expression in *Dilp2GAL4* and *DdcGAL4* domains. (A, C) At 60 hrs and 108 hrs AEL, *itpr^sv35/ug3^* have much less red food in their guts in comparison to *UASitpr^+^/+*;*DdcGAL4/+*;*itpr^sv35/ug3^*, *UASitpr^+^/+*;*Dilp2GAL4/+*; *itpr^sv35/ug3^* and wild-type larvae. (B, D) Spectrophotometric quantification of homogenates from larvae fed yeast paste containing a red dye. Control, *Dilp2GAL4* or *DdcGAL4* rescued larvae ingest significantly more dye than *itpr^sv35/ug3^* (*itpr* mutant) larvae at 60 hrs AEL (*p<0.05; Student's t-test) and at 108 hrs AEL (*p<0.005; Student's t-test). The following number of larvae (n) in batches (N) were assayed for each genotype: At 60 hrs AEL: n = 95 or more, N = 4 for all genotypes; at 108 hrs AEL: for *UASitpr^+^/+;itpr^sv35/^* ^ug3^ L3 n = 46, N = 3; L2 n = 87, N = 3; for all other genotypes n = 100, N = 4 or more. Quantification of cell number in salivary glands from larvae at 60 hrs AEL stained with DAPI to visualize nuclei. *itpr^sv35/ug3^* (*itpr* mutant) in (E) and wild-type are shown in (F). No significant difference (G) was observed in the number of nuclei in *itpr* mutant and wild-type salivary glands. n = 10 salivary glands for each genotype. RT-PCR analysis (H) and quantitative real-time PCR analysis (I, J) revealed significant up-regulation of transcript levels of *d4E-BP* and *dLipase-3* in *itpr^sv35/ug3^* at 60 hrs AEL that can be significantly rescued by *Dilp2GAL4* or *DdcGAL4* driven expression of *UASitpr^+^* (*p<0.005; Student's t-test). Real-time PCR analysis was repeated three times with independently isolated RNA samples for each genotype. Results are expressed as mean±SEM.

The smaller body size observed in *itpr* mutant larvae could be either due to reduced insulin signaling, (as suggested by the rescue of the mutant phenotype by *Dilp2GAL4::UASitpr^+^*) or due to a feeding defect in these mutants or a combination of both. The feeding ability of *itpr* mutants was determined quantitatively by measuring ingestion of colored food (Figure 2). Wild-type larvae in all cases had significant red food in their guts and consequently homogenates derived from these animals show a high absorbance at 520 nm. However, a majority of *itpr^sv35/ug3^* mutant larvae had no or very little food in their gut and thus exhibit reduced absorbance values indicating that they were unable to feed normally (Figure 2A, B, 60 hrs after egg laying (AEL) and Figure 2C, D, 108 hrs AEL). The feeding defect appeared progressive, as many more *itpr^sv35/ug3^* larvae had no food in their gut at 108 hrs than at 60 hrs AEL. Defective feeding behavior could be rescued by expressing *UASitpr^+^* transgene in either the *Dilp2GAL4* or *DdcGAL4* domains (Figure 2A–D). Smaller larvae in *itpr^sv35/ug3^* could also arise as a consequence of fewer cells. However, there was no significant difference in the total number cells in salivary glands from *itpr^sv35/ug3^* larvae as compared to wild-type larvae at 60 hrs AEL (Figure 2E, F and G ).

Starving *Drosophila* larvae up-regulate several molecular markers including *d4E-BP*, a translation repressor and *dLipase-3*, an acid lipase [8], [9]. Unlike *d4E-BP* which is up-regulated by either reduced insulin signaling or starvation, *dLipase-3* is specifically up-regulated upon starvation and not in insulin signaling pathway mutants [10]. Transcript levels of both *d4E-BP* and *dLipase-3* were up-regulated in *itpr^sv35/ug3^* at 60 hrs AEL as determined by reverse-transcriptase-mediated polymerase chain reaction (RT-PCR) analysis (Figure 2H) and quantitative real-time PCR (Figure 2I and J). Expression of the *UASitpr^+^* transgene with either *Dilp2* or *DdcGAL4* reduced this up-regulation (Figure 2H, I and J). The reduction of transcript levels was significantly better with *DdcGAL4* than with *Dilp2GAL4* (Figure 2I and J), similar to the differential rescue of viability shown in Figure 1B. These data strongly suggest that the primary cause of the observed growth defect and lethality in *itpr^sv35/ug3^* larvae is reduced food intake.

### Larger body size in rescued *itpr* mutants is due to a delay in pupation

**Figure 3:**
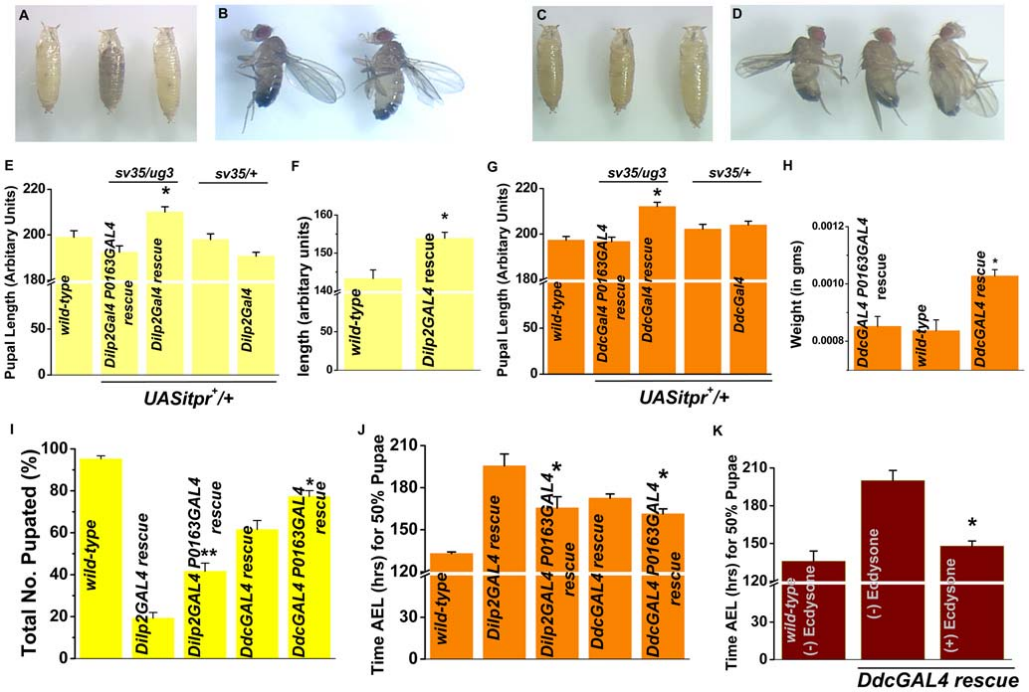
Delayed pupation in *Dilp2GAL4* and *DdcGAL4* rescue conditions results in larger body size. (A) From left to right: *UASitpr^+^/+;Dilp2GAL4/+; P0163GAL4,itpr^sv35/ug3^* (*Dilp2GAL4 P0163GAL4* rescue), wild-type (*Canton-S*) and *UASitpr^+^/+*;*Dilp2GAL4/+*;*itpr^sv35/ug3^* (*Dilp2GAL4* rescue) pupae. (E) Only *UASitpr^+^* expression with *Dilp2GAL4* in *itpr^sv35/ug3^* background causes a significant increase (*p<0.005; Student's t-test) in pupal length as compared to controls of all the indicated genotypes. Pupal length is restored close to wild-type in *Dilp2GAL4::P0163GAL4* rescue condition. (B) From left to right: wild-type and *Dilp2GAL4* rescued flies. (F) Body length of *Dilp2GAL4* rescued flies is significantly more than wild-type flies (*p<0.05; Student's t-test). (C, D) From left to right: *UASitpr^+^/+;DdcGAL4/+;P0163GAL4,itpr^sv35/ug3^* (*DdcGAL4 P0163GAL4* rescue), wild-type (*Canton-S*) and *UASitpr^+^/+*;*DdcGAL4/+*;*itpr^sv35/ug3^* (*DdcGAL4* rescue) pupae (C) and flies (D). (G) Only *UASitpr^+^* expression with *DdcGAL4* in *itpr^sv35/ug3^* background causes a significant increase (*p<0.005; Student's t-test) in pupal length as compared to controls of all the indicated genotypes. Pupal length (G) and adult weight per fly (H) is increased in the *DdcGAL4* rescued condition (*p<0.005; Student's t-test) but is restored close to wild-type in *DdcGAL4::P0163GAL4* rescue condition. n = 10 or more for each individual genotype for (A–G). For (H), the following numbers of male flies (n) in batches of around 3 flies each were weighed for each genotype: *DdcGAL4 P0163GAL4* rescue n = 26, wild-type n = 30 and *DdcGAL4* rescue n = 54. (I) Total number of larvae that undergo pupation is significantly increased on introducing a prothoracic gland GAL4 (*P0163GAL4)* in *DdcGAL4* and *Dilp2GAL4* rescued conditions (*p<0.05, **p<0.005; Student's t-test). (J) Time AEL for 50% pupal formation is significantly reduced with *P0163GAL4* in *DdcGAL4* and *Dilp2GAL4* rescued conditions (*p<0.005; Student's t-test). However the 50% pupation time in all single and double GAL4 conditions remained longer than the 50% pupation time of wild-type. For (I) and (J) 25 larvae in the following number of batches (N) were assayed for pupation for each genotype: wild-type N = 11, *Dilp2GAL4* rescue N = 10, *Dilp2GAL4 P0163GAL4* rescue N = 5, *DdcGAL4* rescue N = 13 and *DdcGAL4 P0163GAL4* rescue N = 9. (K) *DdcGAL4* rescued *itpr^sv35/ug3^* larvae pupated earlier on being fed 20-hydroxyecdysone (∼150 hrs AEL) than larvae without 20-hydroxyecdysone (∼200 hrs AEL) (*p<0.05; Student's t-test). A minimum of 75 animals were screened in batches of 25 each. Differences in pupation rate are not apparent upon 20-hydroxyecdysone feeding in *Dilp2GAL4* rescue animals due to increased lethality in this condition in late third instar larvae. The *DdcGAL4* rescued condition which were not fed ecdysone, pupated at a slower rate than those observed in (J). This is very likely due to differences in culture conditions in the two cases. Results are expressed as mean±SEM.

Since *DdcGAL4* and *Dilp2GAL4* driven expression of *UASitpr^+^* in *itpr^sv35/ug3^* rescues feeding defects and lethality, we expected that larval, pupal and adult size of rescued animals to be similar to wild-type. Analysis of larval size was not possible since it was complicated by the presence of a few larvae in the rescued genotypes, smaller in size than controls. It is very likely that these correspond to animals in which the feeding defect is not completely rescued and which do not pupate finally. Surprisingly, we found a significant increase in pupal size in the *Dilp2GAL4* rescued condition when compared to wild-type animals (Figure 3A and E). Over-expression of the *UASitpr^+^* transgene with *Dilp2GAL4* in wild-type animals did not lead to bigger sized pupae indicating that the larger size is not a consequence of over-active insulin signaling by *UASitpr^+^* expression in IPCs (Figure 3E). A similar increase in body size of individual *Dilp2GAL4* rescued *itpr* mutant flies was also observed (Figure 3B and F). Increased pupal length, body size and adult fly weights were also observed in the *DdcGAL4* rescue of *itpr^sv35/ug3^* (Figure 3C, D, G and H).

A possible reason for the increase in body size and weight of the rescued animals could be a prolonged feeding period as third instar larvae, due to delayed pupation. Increased body size due to a prolonged feeding period is also observed when Prothoracicotrophic Hormone (PTTH) (which stimulates ecdysone synthesis in the prothoraic gland of the ring gland) producing neurons are ablated [11]. In fact, the time taken to pupate by the *UASitpr^+^::Dilp2GAL4* or *UASitpr^+^::DdcGAL4* rescued animals is much more than wild-type animals (Figure 3J). While 50% of wild-type pupae formed by ∼130 hrs AEL, 50% pupation for the Ddc rescued larvae was at ∼170 hours AEL and among Dilp rescued larvae it was ∼195 hrs AEL (Figure 3J). Delays in molting and pupation of *itpr* mutants, independent of their nutritional status, have been reported earlier [3], [12]. These delays could be rescued by feeding 20-hydroxyecdysone to the mutant animals indicating a defect in prothoracic gland function and ecdysone release in *itpr* mutants [3]. To rescue the pupation delay, we expressed the *UASitpr^+^* transgene with a prothoracic gland driver (*P0163GAL4*; [13]). Expression of the *UASitpr^+^* transgene with *P0163GAL4* does not rescue the lethality of *itpr^sv35/ug3^* [4]. Animals expressing the *UASitpr^+^* transgene simultaneously with *P0163* and either the *Dilp2* or *DdcGAL4* had more surviving pupae (Figure 3I) and pupated ∼12–20 hours earlier than when expression was driven only by *Ddc* or *Dilp2GAL4* (Figure 3J). Pupal and adult fly sizes of the double GAL4 rescued animals were comparable to that observed for wild-type animals (Figure 3A, C, D, E, G and H). The time to reach 50% pupation in *DdcGAL4* rescued animals was significantly reduced when they were fed with 20-hydroxyecdysone (Figure 3K). Ecdysone feeding of *Dilp2GAL4* rescued animals caused significant lethality and hence the time taken to pupate could not be measured accurately. Feeding ecdysone to wild-type larvae does not cause a similar speed-up of pupation but is known to reduce viability [14]. These results support an independent requirement of InsP_3_R activity in *Drosophila* prothoracic glands for the synthesis and/or release of ecdysone [3]. A role for intracellular Ca^2+^ release in ecdysone and steroid biogenesis has been previously proposed for Manduca [15] and the mammalian adrenal glands [16], [17] respectively.

### Relation between the *Dilp2GAL4* and *Ddc* domains

**Figure 4:**
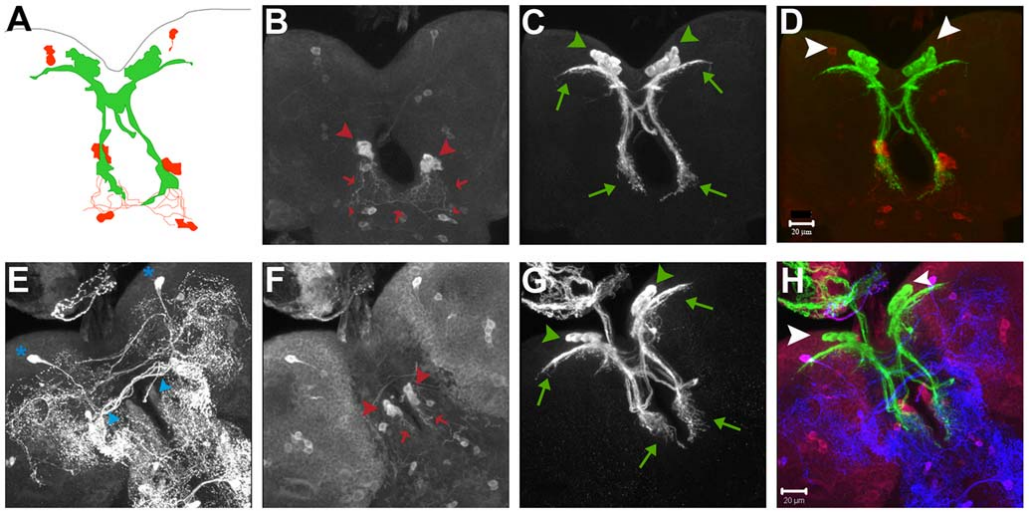
Ddc and serotonin labeled cells in larval brains do not overlap with IPCs. (A) A schematic drawing depicting a third instar larval brain with the relative positions of the IPCs and their processes (in green) and the Ddc labeled cells and their processes (in red). The cellular processes from the two domains seem to intermingle in the sub-esophageal ganglia region. (B–H) Three-dimensional projections of confocal Z-stacks of a wild-type *Drosophila* larval brain from a wandering third instar larva expressing mCD8GFP with *Dilp2GAL4* and immunostained with anti-serotonin antibody (E), anti-Ddc antibody (B, F) and anti-GFP antibody, (C, G). (D) is a merge of (B) and (C) while (H) is a merge of (E),(F) and (G). In (D) and (H), anti-Ddc staining is in red and anti-GFP in green while anti-serotonin is blue in (H). Red arrowheads in (B, F) indicate Ddc stained cells in the sub-esophageal ganglia that lie in close proximity to IPC projections (bottom green arrows in C, G). Smaller red arrowheads indicate cells which send out processes (marked with red arrows) that seem to intermingle with these IPC projections. Green arrowheads in (C, G) mark the IPCs in the two brain lobes. Green arrows indicate the projections of the IPCs towards the lateral protocerebrum (top green arrows) and sub-esophageal ganglion (bottom green arrows). Ddc marked cells (indicated by big red arrowheads in B, F) stain with the anti-serotonin antibody (E, marked by blue arrowheads), but have lesser serotonin staining than some neighboring cells (for example, cells in the lateral protocerebrum indicated by blue asterisk in E). Scale bars B–H 20 µm.

The simplest explanation for rescue of *itpr* mutant phenotypes by restoring itpr function in the IPCs and aminergic neurons is that an overlap exists between the two domains. In order to determine this, a membrane bound GFP (*UASmCD8GFP*) was expressed with *Dilp2GAL4* and larval brains of these animals were stained with an anti-Ddc antibody [18]. In the third instar larval brain, the IPCs consist of two bilaterally symmetric clusters of neurosecretory cells in the pars intercerebralis region of the protocerebrum (green arrowheads in Figure 4C and G) [19]. These did not stain with the anti-Ddc antibody (white arrowheads in Figure 4D and H). The IPC clusters extend processes that terminate at the lateral protocerebrum and sub-esophageal ganglion (green arrows in Figure 4C and G) [6]. We observed a pair of anti-Ddc stained cell clusters, each consisting of about four cells, located medially in the sub-esophageal region (red arrowheads in Figure 4B and F). Ddc labeled processes that emerge from these cells lie in close proximity to the processes originating from the IPCs (red arrows in Figure 4B and F). Though *DdcGAL4* expresses in both serotonergic and dopaminergic neurons, *itpr* mutant phenotypes are not rescued by expression of *UASitpr^+^* in the dopaminergic domain (with the *THGAL4* [20], unpublished data) suggesting that the aminergic domain rescue of *itpr* mutants is through serotonergic neurons in the context of the phenotypes under study. In order to determine whether anti-Ddc stained cell clusters (red arrows in Figure 4B and F) produce serotonin or dopamine, these brains were stained with an anti-serotonin antibody. A previous report has found strict segregation of serotonin and dopamine producing cells [18]. However, we observed differential levels of serotonin in cell bodies of larval brains. The cluster of Ddc labeled cells in the sub-esophageal ganglion seemed to contain lower levels of serotonin as compared with other cells that had higher serotonin staining (compare cells indicated with blue arrowhead vs asterisk in Figure 4E). Additional segmentally organized cells that were serotonin and Ddc positive were observed just posterior to this cluster of cells (small red arrowheads in Figure 4B) and these also gave rise to processes (red arrows in Figure 4B) that terminated in the sub-esophageal ganglion, once again in close proximity to processes from the IPC clusters (green arrows in Figure 4C). Numerous serotonergic varicosities were present on the processes emanating from the IPC clusters that terminate at the lateral proto-cerebrum and sub-esophageal ganglion. Interestingly, the subesophageal ganglion region has been implicated in feeding and taste responses, as gustatory sensory neurons and hugin neurons (that are known to modulate feeding behavior) project to this region [21]. We find that both IPCs and Ddc positive neurons also project to the sub-esophageal ganglion, suggesting the possibility of neuronal communication with the gustatory and hugin neurons to regulate feeding. Moreover, in each brain lobe, many serotonergic varicosities were observed in close proximity to the main cell bodies of the IPCs, as has been observed earlier [22]. No overlap of Ddc labeled cells and the GFP marked IPCs was observed in the ventral ganglia.

**Figure 5:**
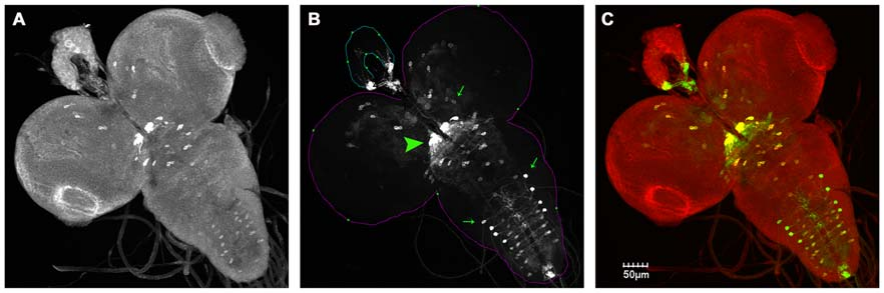
Overlap of *DdcGAL4* and Ddc labeled cells in larval brains. Three-dimensional projections of confocal Z-stacks of a wild-type *Drosophila* 3^rd^ instar larval brain (A–C) expressing mCD8GFP with *DdcGAL4* and immunostained with an anti-Ddc antibody (A) and anti-GFP antibody (B). A majority of *DdcGAL4::UASmCD8GFP* labeled cells overlap with those stained with the Ddc antibody, though there are some cells in both cases that do not overlap (green arrows in B). These do not appear in the region of the IPCs. Green arrowhead in (B) indicates *DdcGAL4::UASmCD8GFP* expression in Ddc stained cells in the sub-esophageal ganglia that lie in close proximity to IPC projections. Scale bars A–C, 50 µm.

To confirm that the Ddc antibody being used provides an accurate representation of the *DdcGAL4* domain, *DdcGAL4::UASmcCD8GFP* brains were stained with the Ddc antibody in the larval stages (Figure 5A). These experiments showed that though a majority of *DdcGAL4* labeled cells (Figure 5B) stained with the Ddc antibody, there are some cells that do not overlap (green arrows in Figure 5B). However, these do not appear in the region of the IPCs. Strong expression of the *DdcGAL4* was observed in the cluster of anti-Ddc labeled cells in the sub-esophageal ganglion (green arrowhead in Figure 5B). These experiments indicate the absence of any detectable overlap between the *Dilp* and the *DdcGAL4* domains in larval brains.

### Ubiquitous and tissue specific knockdown of *itpr* by RNA interference

**Figure 6:**
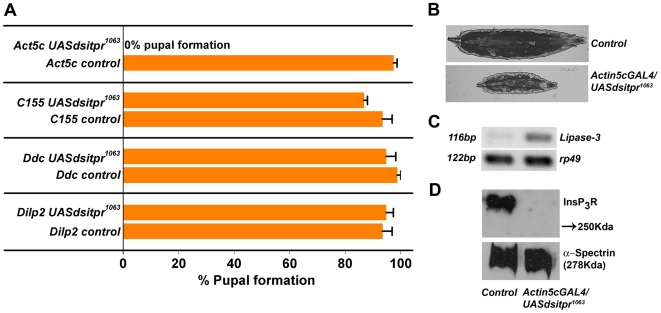
Ubiquitous but not tissue specific knockdown of *itpr* recapitulates *itpr^sv35/ug3^* phenotypes. (A) *Act5c dsitpr* larvae (*Act5cGAL4*/*UASdsitpr^1063^*) did not undergo pupation and died as 3^rd^ instars. The number of pupae formed in *Dilp2 dsitpr* (*Dilp2GAL4/UASdsitpr^1063^*; *UASdicer/+*), *Ddc dsitpr* (*DdcGAL4/UASdsitpr^1063^*; *UASdicer/+*) and *C155 dsitpr* (*Elav^C155^GAL4; UASdsitpr^1063^/+;UASdicer/+*) was similar to controls (not significant; p>0.05; Student's t-test). GFP positive larvae were used as controls in each RNAi experiment. Three batches of 25 2^nd^ instar larvae were screened for each of the indicated genotypes. Results are expressed as mean±SEM. (B) Significant reduction in larval size was observed in *Actin5cGAL4/UASdsitpr^1063^* 3^rd^ instars (∼120 hrs AEL) compared to controls (*Actin5cGAL4 or UASdsitpr^1063^*/CyoG). (C) RT-PCR gel with up-regulation of d*Lipase-3* transcript levels in RNA isolated from *Actin5cGAL4/UASdsitpr^1063^* larvae (∼ 85 hrs AEL) compared to RNA from controls. (D) A western blot with reduced InsP_3_R (280Kda) protein levels in lysates from *Actin5cGAL4/UASdsitpr^1063^* 3^rd^ instar (∼120 hrs AEL) larvae as compared to lysates from controls. Equal levels of the loading control α-spectrin (278Kda) confirm that similar quantities of protein lysates were loaded in each lane.

Absence of a visible overlap between the *Dilp* and *Ddc* domains raises the question of the extent and mode of contribution of each domain to the rescue of *itpr* mutant phenotypes. To assess individual contributions, we obtained several RNA interference (RNAi) lines for the *itpr* gene and measured their effect on larval viability by ubiquitous expression with an *Actin5cGAL4.* Amongst the *dsitpr* lines tested, one line referred to as *UASdsitpr^1063^*, does not survive beyond the larval stages on expression with the *Actin5cGAL4* line (Figure 6A). Larvae of the genotype *Actin5cGAL4/UASdsitpr^1063^* were significantly smaller in size than controls of the genotype *Actin5cGAL4* or *UASdsitpr^1063^*/CyoGFP at ∼ 120 hrs AEL (Figure 6B). They appeared similar in size to controls at an earlier time point (∼ 85 hrs AEL) when they had significantly higher levels of *dLipase-3* transcripts, indicating that feeding defects preceded changes in size and subsequent lethality (Figure 6C). There is a near complete absence of the InsP_3_R in protein lysates of *Actin5cGAL4/UASdsitpr^1063^* 3^rd^ instar larvae (Figure 6D). These larval phenotypes are analogous to those observed in *itpr^sv35/ug3^* and re-emphasize the importance of InsP_3_R activity for feeding and larval viability. Expression of *dsitpr^1063^* with the pan-neuronal GAL4 (*Elav^c155^*) or with either *Dilp2GAL4* or *DdcGAL4* had no significant effect on larval viability or size (data not shown) as judged by the number and size of pupae formed (Figure 6A). This was despite enhancing RNAi by introducing a *UASdicer2* transgene [23] in the background. These results suggest that *itpr* knockdown in neuronal or sub-neuronal domains is insufficient for phenocopying larval *itpr* mutant phenotypes. More complex interpretations are also possible (see discussion).

## Discussion

In this study we find that the loss of viability in *itpr* mutant larvae is preceded by feeding deficits. Viability, size and feeding deficits can all be rescued significantly by *itpr^+^* expression in the IPCs as well as in aminergic neurons. The two cellular domains do not exhibit a visible overlap. Thus the rescues are not mediated by a shared neuronal subset. However, the two domains are closely apposed suggesting that they could interact. While ubiquitous depletion of *itpr* by dsRNA can phenocopy strong *itpr* mutants, depletion of *itpr* in IPCs or aminergic neurons produces no obvious phenotype, indicating that InsP_3_R-mediated calcium release has a modulatory role in these neuronal domains.

### InsP_3_ signaling in energy metabolism, growth and viability

A role for InsP_3_ signaling in regulating metabolism and growth in mammalian systems has been previously suggested from studies of InsP_3_R mutant mice [24], [25]. Body mass and overall brain sizes were found to be reduced by half in weight in homozygous InsP_3_R type 1 knock out mice as compared to control mice [24]. These mice gradually become emaciated and died by postnatal day 25 or 26 [26]. Interestingly, homozygous *opisthotonos* pups, that have a functionally altered InsP_3_R type 1, are also smaller than their littermates [25]. Moreover, InsP_3_R type 2 and type 3 double mutants appeared similar to their control littermates at birth, but subsequently started losing weight and died within the 4^th^ week of age when fed on dry food due to a defect in the secretion of saliva [27]. These results mirror the growth defects and lethality we observe in *itpr^sv35/ug3^* organisms and by the ubiquitous knockdown of *itpr*. However, neither pan-neuronal knockdown of *itpr* nor specific knockdown in IPCs or aminergic cells results in larval lethality though expression of *itpr^+^* in the same domains is able to rescue lethality observed in *itpr^sv35/ug3^* ([4] and this study). This difference in the expected phenotypes probably arises due to a difference in the nature of rescue experiments compared to RNAi experiments. Expression of the *itpr^+^* gene occurs in multiple larval tissues including the central nervous system [12]. Therefore larval lethality in *itpr^sv35/ug3^* is possibly a combination of both neuronal and non-neuronal perturbations. This is supported by the strong lethality observed on ubiquitous knockdown of *itpr*. Reduction of InsP_3_R in either the neuronal or a sub-neuronal domain would then be insufficient for inducing lethality. Restoration of *itpr^+^* in the neuronal domain or specifically in IPCs or aminergic neurons might rescue lethality by non-cell autonomous mechanisms, such as modulating the release of either DILPs or serotonin. In this condition the system may not restore to a wild-type state at every level but instead achieve a new stable state in which a wild-type output is preserved [28]. Similar circuit outputs can be generated by multiple mechanisms [29], making it plausible that different stable states are achieved in the *DdcGAL4* and *Dilp2GAL4* rescue conditions. It is also conceivable that the native function of the InsP_3_R in IPCs and aminergic cells can take place with extremely low levels of protein that persists in the RNAi knockdown condition. The reduced sensitivity of the anti-dInsP_3_R for immuno-histochemistry prevents a direct assessment of this last possibility.

InsP_3_Rs are present in mammalian pancreatic beta cells that release insulin and InsP_3_ has been shown to cause release of calcium from intracellular stores in these cells [30], [31]. InsP_3_R is postulated to participate in the calcium oscillatory capacity of these cells in response to glucose which is required for insulin vesicle secretion [32]. Stimulation of mouse primary beta cells or MIN6 insulinoma cells with glucose led to oscillatory InsP_3_ generation that was tightly coupled with calcium increase, but was found not to be the driving force for the calcium oscillations that led to insulin release [33], [34]. In addition to glucose, insulin secretion from the beta cells is also modulated by coordinated inputs from several gut hormones and neurotransmitters [35]. Among these, acetylcholine plays a prominent role by binding to the muscarinic cholinergic receptors which activate the PLC-InsP_3_ pathway to elevate cytosolic calcium and facilitate insulin vesicle exocytosis [36]. Interestingly, islets from mutant mice selectively lacking the M_3_ muscarinic receptor in pancreatic beta cells have a dramatic decrease in agonist induced inositol phosphate production and insulin secretion [37]. These studies suggest a modulatory role for InsP_3_R activity in regulating insulin secretion form mammalian pancreatic beta cells. In this study, we find that restoring InsP_3_R activity in the IPCs of *itpr* mutant larvae rescues larval lethality, growth and feeding to a significant extent. However, *itpr* knockdown specifically in IPCs does not result in the converse phenotypes suggesting that reduced InsP_3_R activity does not impair DILP secretion and argues for a modulatory role for InsP_3_R in *Drosophila* IPCs, similar to the scenario in mammalian pancreatic beta cells.

### Regulation of feeding and growth by InsP_3_, insulin and serotonin signaling

In *Drosophila*, hyperactivation of the Insulin Receptor /PI3 Kinase signaling as well as over-expression of *dFOXO*, a direct mediator of insulin signaling, alters larval feeding behavior [38]–[40]. Serotonergic innervation is found in the *Drosophila* larval feeding apparatus [41] and decreased feeding behavior is observed in null mutants of neuronal *Tryptophan hydroxylase* gene [42], the rate limiting enzyme in serotonin synthesis. Since, expression of the InsP_3_R in either IPCs or Ddc cells restores normal feeding behavior in *Drosophila*, the existence of an evolutionarily conserved system of energy intake and utilization involving insulin and serotonin is possible [43], [44]. A Iink between InsP_3_R function and the control of feeding has also been suggested in *Caenorhabditis elegans* [45].

The absence of any cellular overlap between aminergic and DILP producing neurons suggests that these domains regulate feeding and growth through secreted serotonin and DILPs and thus communicate with each other or influence a common subset of downstream cells by binding of serotonin and DILP to their cognate receptors. High levels of *Drosophila* Insulin Receptor (*dIR*) mRNA are present in the larval and adult nervous system [46] and dIR protein has been localized to the larval brain [47] and in the fat body surrounding the adult brain [48]. Serotonergic varicosities are thought to engage primarily in volumetric type neurotransmission in which neurotransmitter is released for distribution over a region of neuropil containing many target synapses and therefore serotonergic varicosities often do not have post-synaptic partners [49], [50]. *Drosophila* serotonin receptors 5-HT1BDro (d5-HT1B) and 5-HT2Dro have been observed in larval and adult brains [51], [52]. Interestingly, the Gq/InsP_3_-coupled 5-HT_2_CR is a key mediator of the serotonergic suppression of feeding and agonists of this receptor were found to significantly improve glucose tolerance and reduce plasma insulin in murine models of obesity and type 2 diabetes [53]. Unlike mammalian systems, ATP-sensitive K^+^ channels that respond directly to glucose levels and signal insulin release are not present on *Drosophila* IPCs [6]. This implies that there might be other signaling mechanisms that integrate environmental, nutritional and physiological information to modulate DILP secretion from the IPCs and serotonergic signaling working through the Gq/InsP_3_ pathway could be one such mechanism [22], [54].

## Materials and Methods

### Drosophila Strains

*itpr^sv35/ug3^* is a heteroallelic combination of single point mutants in the *itpr* gene that were generated in an EMS (ethyl methanesulfonate) screen. Detailed molecular information on these alleles has been published [4]. The embryonic wild-type *itpr* cDNA (*UASitpr^+^*) [12] was used for rescue experiments. *itpr* RNAi experiments were done with the *UASdsitpr* (1063R-2) line from the National Institute of Genetics Fly Stock Center, Japan. The *Dilp2GAL4* strain was from Dr. E. Rulifson [6]; *DdcGAL4* [55], *P0163GAL4* [56], *Actin5cGAL4 (4414)*, *Elav^C155^GAL4* and *UASdicer(III) (24651)* were obtained from the Bloomington Stock Centre. The other fly strains used were generated by standard genetic methods using individual mutant and transgenic fly lines described above.

### Larval staging and lethality measurements

To obtain molting profiles, staging experiments were performed with minor modifications as described previously [4]. Timed and synchronized egg collections were done for a period of 8 hrs at 25°C and the cultures were allowed to grow further at this temperature. Larvae of the desired genotype were selected at 56–64 h AEL and transferred into vials of cornmeal medium lacking agar. These larvae were grown at 25°C and screened at appropriate time points, for number of survivors and their stage of development. For each time interval, a minimum of 75 animals were screened in batches of 25 each.

### Feeding Assay

Yeast paste containing red dye (Carmoisine Red; Anand Dyes and Co. Ltd., Mumbai, India) was placed centrally on 90mm petri dishes plated with 2% agar in Phosphate Buffered Saline (PBS). Larvae of the appropriate age and genotype were placed on red yeast paste and allowed to feed for 4 hrs (at 60 hrs AEL) or 2 hrs (at 108 hrs AEL). After feeding, each group of larvae were washed in distilled water, dried on blotting paper and placed in 1.5 ml tubes and immediately frozen in liquid nitrogen. Larvae were then homogenized in PBS, centrifuged at 14 *g* for 5 minutes and the supernatant was transferred to a fresh tube. The supernatant was mixed with PBS and the Abs_520_ read.

### RT-PCR and real time analysis

2^nd^ instar larvae of the indicated genotypes were selected at 56–64 h AEL and snap frozen in liquid nitrogen. Total RNA was extracted with *Trizol* Reagent (Invitrogen) according to the manufacturer's protocol. Approximately 1 µg of purified total RNA was used for reverse transcription reactions. cDNA was generated using gene specific primers and MMLV reverse transcriptase (Invitrogen) at 42°C for 1 hour. Polymerase chain reactions (PCRs) were performed using cDNA as template in a 25 µl reaction. *rp49* gene primers were used for internal normalization of every batch of RNA. The same sense and antisense primers were used for RT-PCR and Realtime PCR. Quantitative realtime PCRs were performed on the Rotor-Gene 3000 (Corbett Research, Australia) operated with Rotor Gene software version 6.0.34 using SYBR^®^ Green JumpStart™ *Taq* ReadyMix (Sigma). Experiments were performed with *rp49* and the gene of interest, using serial dilutions (1∶100, 1∶1000 and 1∶10,000) of the cDNA preparation. The experiment was repeated three times with independently isolated RNA samples. Cycling parameters were 95°C for 10 min, 45 cycles of 95°C for 20 s and 53°C (for *rp49*) and 55°C (for *4E-BP* and *Lipase-3*) for 30 s, 72°C for 30 s, then 1 cycle of 72°C for 5 min and hold at 50°C for 1 min. The fluorescent signal produced from the amplicon was acquired at the end of the polymerization step at 72°C. A melt curve was also performed after the assay to check for specificity of the reaction. Amplification primers were as follows:

*rp49*, 5′ATGACCATCCGCCCAGCATAC; 3′TTACCTCGTTCTTCTTGAGAC; *4E-BP*, 5′CATGCAGCAACTGCCAAATC;3′CCGAGAGAACAAACAAGGTGG ; *Lipase-3*, 5′TGAGTACGGCAGCTACTTCCCT; 3′TCAACTTGCGGACATCGCT

The fold change in the mutant's target gene cDNA relative to wild-type *Drosophila* (*Canton S)* was determined by the comparative ▵▵C_t_ method [57]. In this method the fold change = 2^−▵▵C^_t_ where ▵▵Ct = (*C*_t(target gene)_−*C*_t(rp49)_)_mutant_ −(C_t(target gene)_−C_t(rp49)_)_Wild type_.

Amplification primers used for the experiment in Figure 6 for *rp49* were as follows: 5′CGGATCGATATGCTAAGCTGT; 3′ATGCCTAGCTTGTTCGCG.

### Immunohistochemistry

Immunohistochemistry was performed on *Drosophila* larval brains expressing a membrane bound GFP (*UASmCD8GFP*) with the *Dilp2GAL4* or *DdcGAL4* that were fixed in 4% paraformaldehyde for 30 minutes. The following primary antibodies were used - rat anti-Ddc (1∶400; provided by Dr. J. Hirsh), rabbit anti-GFP antibody (1∶10,000; Molecular Probes) and monoclonal anti-5-HT antibody (1∶50; NeoMarkers, Fremont, CA). The following fluorescent secondary antibodies were used at a dilution of 1∶400 - anti-rabbit Alexa Fluor 488 and anti-rat Alexa Fluor 633 (Molecular Probes, Eugene, OR) and anti-mouse Rhodamine Red X (Jackson Laboratories). Confocal analysis was performed on a Zeiss LSM 510 Meta microscope (Carl Zeiss Micro Imaging, Inc.) or an Olympus Confocal FV1000 microscope using 20X 0.9 N.A. or 63X 1.4 N.A. objectives. Confocal data were acquired as image stacks of separate channels and combined and visualized as three- dimensional projections using the LSM5 version 3.2/SP2 software or FV10-ASW 1.3 viewer.

Salivary glands derived from 60 hr AEL larvae were dissected in PBS, fixed in 4% paraformaldehyde, and stained with DAPI to visualize nuclei. Images were acquired at different focal planes and the total number of nuclei per salivary gland was counted.

### Western Blots

Protein extracts from 3^rd^ instar larvae of the indicated genotype were run on a 5% SDS-polyacrylamide gel and transferred to nitrocellulose membrane by standard western blotting protocols. The affinity purified anti-DInsP_3_R rabbit polyclonal antibody (IB-9075) raised against KLH-conjugated peptide CEQRKQKQRLGLLNTTANSLLPFQ derived from the DInsP_3_R sequence [58] was used at a dilution of 1∶300. The mouse α-spectrin antibody (1∶50 dilution, DSHB) was used as a loading control. Total protein estimation using the BCA (Bicinchoninic Acid) Kit (Sigma Aldrich) was performed to confirm that equal quantity of protein was loaded. Secondary antibodies conjugated to horseradish peroxidase were used, and the detection of protein in the blot was done by addition of a chemiluminescence substrate from Pierce (catalog #34075; Rockford, IL).

### Statistical analysis

Computation of means, SEM, and Student *t-*tests was performed using Origin software (Origin Lab, Northampton, MA) in all experiments.
